# Risk Factors of Peripheral Vascular Disease in Diabetes Mellitus in Abbottabad, Pakistan: A Cross-Sectional Study

**DOI:** 10.7759/cureus.17556

**Published:** 2021-08-30

**Authors:** Abdul Majid Khan, Petras Lohana, Priyanka Anvekar, Syed Hassan Mustafa, Ramesh Kumar, Adnan LNU, Pushpa Bhimani, Syed R Ali, Arti LNU, Syed Hamad Ali Shah

**Affiliations:** 1 Assistant Professor, Department of Medicine, Ayub Medical College, Abbottabad, PAK; 2 Internal Medicine, Liaquat University of Medical and Health Sciences Hospital, Karachi, PAK; 3 Medicine and Surgery, Mahatma Gandhi Mission Medical College and Hospital, Mumbai, IND; 4 Consultant, Department of Medicine, Ayub Medical College, Abbottabad, PAK; 5 Internal Medicine, Civil Hospital Karachi, Karachi, PAK; 6 Assistant Professor, Department of Internal Medicine, Ayub Medical College, Abbottabad, PAK; 7 Obstetrics and Gynecology, Civil Hospital Karachi, Karachi, PAK; 8 Internal Medicine, Dow University of Health Sciences, Civil Hospital Karachi, Karachi, PAK; 9 Bachelor of Medicine and Bachelor of Surgery (MBBS), Liaquat University of Medical and Health Sciences, Kunri, PAK; 10 Internal Medicine, Frontier Medical and Dental College, Abbottabad, PAK

**Keywords:** diabetes mellitus, peripheral vascular disease, smoking, hypertension, hypercholesterolemia, obesity

## Abstract

Introduction

Diabetes mellitus (DM) is a significant and common risk factor for the development of peripheral vascular disease (PVD). Peripheral vascular disease is the atherosclerotic narrowing of peripheral arteries and has a high prevalence among patients with diabetes.

Material and methods

A cross-sectional study was conducted in the Department of Medicine of Ayub Teaching Hospital, Abbottabad. A total of 271 diagnosed diabetic patients aged 40 years or above were included in the study. Ankle-brachial pressure index (ABPI) was measured using a hand-held Doppler device and sphygmomanometer. An ABPI < 0.9 was taken to be abnormal. The risk factors were noted through history taking, physical examination, and appropriate investigations.

Results

Our study sample included 271 patients. A hundred and forty-five (53.5%) of them were males, and 126 (46.5%) were females. Fifty-three (19.9%) out of 271 patients had peripheral vascular disease. The prevalence of peripheral vascular disease was stratified among smoking (p=0.00), hypertension (p=0.00), obesity (p=0.004), and hypercholesterolemia (p=0.005) to determine if there was any association between these and peripheral vascular disease. A p-value less than 0.05 was taken to be significant.

Conclusion

This study showed a significant association between PVD and smoking, hypertension, hypercholesterolemia, and obesity.

## Introduction

Diabetes mellitus (DM), or simply diabetes, is one of the significant health hazards worldwide [1]. Diabetes is deﬁned as a condition characterized by abnormally high blood sugar levels and leads to many complications affecting almost every system of the body. Among the common complications, the most dangerous include coronary artery disease (CAD) and cerebrovascular disease. Diabetes generally results in early death from CAD [2].

According to the International Diabetic Federation (IDF), in the year 2019; more than 463 million people were suffering from DM around the world, and this figure is expected to rise to 700 million by 2045 [3]. In Pakistan, it is estimated that 16.98% of all adults are suffering from DM [4].

Among the long-standing complications, peripheral vascular disease (PVD) is one of them and is associated with more deaths and amputation [5]. PVD, which is also known as peripheral artery disease (PAD), is the atherosclerotic narrowing/occlusion of vessels other than the coronary arteries or the arterial vasculature of the brain [6]. Most patients with PVD have generalized vascular atherosclerosis, and many of these patients die from cardiac and cerebrovascular-related events.

PVD has become a global health problem affecting more than 200 million people around the world [7]. One reason for high prevalence is that in many cases, PVD is silent and less than 20% of patients report typical symptoms of PAD [7,8]. Due to peripheral neuropathy, the prevalence of asymptomatic PVD is even higher in diabetic patients, estimated to be almost one-third of all diabetic patients [7]. The incidence of PVD and neuropathy has not been measured in Abbottabad until now, making the study an important topic of discussion.

Various risk factors have been described for the increased predisposition to the development of PVD in DM. The common factors observed are hypertension (80.9%), hypercholesterolemia (74.0%), smoking (22.8%), obesity (49.6%), and dyslipidemia (50%) [9-11]. Other factors found are the duration of DM, degree of hyperglycemia, increasing age, male gender, increased serum lipoprotein (a) levels, insulin resistance, increased serum ﬁbrinogen levels, micro-albuminuria and increased levels of intercellular adhesion molecules [12].

This study aimed to report the relationship of different risk factors with silent PVD and assess its magnitude since no local data is available. In addition, the use of ankle-brachial pressure index (ABPI) was evaluated in the early diagnosis of asymptomatic PVD in people with diabetes.

## Materials and methods

This cross-sectional descriptive study was conducted from September 1, 2016 to August 31, 2017, at the Department of Medicine, Ayub Teaching Hospital, Abbottabad. Our sample included 271 patients enrolled through convenient sampling (non-probability sampling). Only patients suffering from DM for the last five years or more who did not exhibit symptoms of peripheral vascular disease and were aged 40 and above were included in the study. Similarly, those patients who were on vasodilators, lipid-lowering agents, or antiplatelet therapy or patients presenting with hypovolemia or renal failure requiring dialysis were excluded from the study to control confounding. Those diabetic patients with impaired ankle-brachial index (ABI), i.e., < 0.9, as measured by the radiologist, were considered to have peripheral vascular disease. The radiologist was kept blind and used a pulse wave Doppler (model - LOGIQ™ P9 manufactured by GE Healthcare). The following common risk factors were considered as smoking (at least one pack/day for three or more years) [13], hypertension (diagnosed case of hypertension for at least five years), hypercholesterolemia (serum cholesterol levels > 6 mmol/L based on lab values), obesity [body mass index (BMI), i.e., weight in kg/height in m2 > 27 at presentation].

Our study was approved by the chairperson of the Institutional Review Board (IRB) and hospital ethical committee at Ayub Teaching Hospital, Abbottabad under the IRB number ATH/EC- 9/16(1). Informed consent was taken from the patients, and the purpose and details of the research work were explained to them. All patients were then subjected to detailed history and examination followed by relevant investigations.

ABI was measured using a hand-held Doppler device and sphygmomanometer. Systolic blood pressure in both arms (brachial arteries) was checked after instructing the patients to lie in the supine position for 10 minutes. A higher value of the two forces was considered the denominator. Next, the blood pressure in the posterior tibial artery and dorsalis pedis artery was measured in both legs. The higher of the two blood pressures were taken as the numerator for each foot. ABI was calculated separately for each leg as the ratio of the systolic pressure in the dorsalis pedis or posterior tibial artery (whichever was higher) to the pressure in the brachial artery.

ABPI = PLeg/Parm

PLeg is the systolic pressure in each leg measured either in the posterior tibial artery or dorsalis pedis artery - whichever was higher & Parm is the higher of the systolic pressure in both arms (brachial arteries).

An ABPI < 0.9 was considered abnormal. The patients were divided into two groups (those having impaired ABPI and those with normal ABPI). The associated risk factors, i.e., smoking, hypertension, obesity, and dyslipidemia, were noted through history, examination, and investigation, respectively, in both groups. All the information was recorded on the pre-designed pro forma.

Data were analyzed statistically using the software SPSS Inc. Released 2007. SPSS for Windows, Version 16.0. Chicago, SPSS Inc. Mean was the measure for quantitative variables like age, duration of diabetes, BMI, blood pressure, and ABPI. Categorical variables like PVD were described as frequencies and percentages. PVD was stratified among diabetic patients to see the effect modifications. The chi-square test at a 5% significance level was used to show an association of risk factors.

## Results

A total of 271 patients were studied in this study. The oldest patient was 80 years old, while the youngest patient was 40 years, with a mean of 56.15. A hundred and forty-five (53.5%) patients were men and 126 (46.5%) were women. Some other characteristics are shown in Table 1.

**Table 1:** Summary of Quantitative Variables (n = 271)

	Minimum	Maximum	Mean
Age of Patients	40	80	56.15
Duration of Diabetes	5	35	13.44
Body Mass Index	19	38	25.41
Systolic Blood Pressure	80	180	125.52
Diastolic Blood Pressure	60	120	81.48
Ankle Brachial Pressure Index	0.63	1.45	1.04
Serum Cholesterol Levels	3.60	7.40	5.33

PVD was diagnosed in 53 (19.6%) patients out of 271. Among the 53 patients, eight (15.09%) had type 1 DM, and 45 (84.91%) had type 2 DM. The total number of patients with type 1 DM was 54 (19.9%), and the total number of patients with type 2 DM was 217 (80.1%). A significant association was noted between PVD and smoking (p-value 0.00), hypertension (p-value 0.00), hypercholesterolemia (p-value 0.005), and obesity (p-value 0.004). No significant association between type of diabetes and PVD was found (p-value 0.326).

**Table 2:** Prevalence of peripheral vascular disease in smokers, hypertensive, obese, and hypercholesterolemia patients

Characteristic	Peripheral vascular disease Present	Peripheral vascular disease Absent	Total	p-value
SMOKING STATUS				
Smoker	24	35	59	0.000
Non-smoker	29	183	212	
Total	53	218	271	
HYPERTENSIVE STATUS				
Hypertensive	33	62	95	0.000
Non Hypertensive	20	156	176	
Total	53	218	271	
SERUM CHOLESTEROL STATUS				
Hypercholesterolemia	20	43	63	0.01
Normal cholesterol	33	175	208	
Total	53	218	271	
OBESITY STATUS				
BMI less than 27	28	160	188	0.004
BMI 27 or more	25	58	83	
Total	53	218	271	

## Discussion

Literature shows that PVD is much more prevalent in diabetic patients compared to the general population. Its prevalence in the type 2 diabetic population of Pakistan is reported to be 16.97% in 2018 [4].

Likewise, there are contradicting reports about the association of PVD with diabetes mellitus. On the one hand, reports suggest no relationship between the type of DM and peripheral vascular disease. However, some research work has suggested that PVD is more prevalent in type 2 DM [14]. This may be because there are more patients with type 2 DM than with type 1 DM.

Similarly, in our study, no significant association was found between the type of diabetes and PVD. However, we found that the association was statistically significant between PVD and smoking, hypertension, hypercholesterolemia, and obesity (p values 0.00, 0.00, 0.005, and 0.004, respectively). Our study is following Wilcox et al. However, Wilcox et al. reported that smoking had the strongest association while obesity had the slightest association with PAD among the said factors [15].

There are variable reports of the prevalence of PVD in Pakistan. The prevalence of PVD in this study was 19.9%. A report from Bahawal Victoria Hospital, Bahawalpur, shows a prevalence of 1.2% for PVD [16]. There is a massive difference between the results of this study and the study at Bahawalpur. The researchers had included only 20 patients already diagnosed with PVD. They reported that the prevalence of PVD was 1.2% and that for 90% of the cases of PVD, smoking was the major predisposing factor. Although the study mentions that 60% of the patients were older than 40 years of age and about 90% of the patients were male, it doesn't say these as the risk factors for PVD [16]. In contrast, our study had recruited 271 patients, and PVD was diagnosed in 53 (19.9%) of these patients. PVD was found to have a significant association with hypertension, smoking, hypercholesterolemia, and obesity in these patients.

Similarly, Zia et al. 2007 reported that the frequency of PVD was 35% [17]. They said that PVD was more common in males and after 50 years of age. They did not attempt to find its association with other risk factors. Although increasing age and male sex have been reported to be risk factors for PVD, this study focused on finding the association with other risk factors. The results reported by Zia et al. are in contrast to the effects of research by Akram et al. [18], who noted no significant difference between males and females when it came to acquiring PVD. They found an association between obesity and higher waist circumference and PVD. They reported that there was no association between the duration of DM or cigarette smoking and PVD. The results of this study partially conform to those written by Akram et al. This study found a significant association between cigarette smoking, obesity, hypertension, and hypercholesterolemia. 

Another research published in 2012 by Ali et al. reported that the prevalence of PVD was more (39.28%) in patients with DM than in the general population. Among the diabetic population, it was more prevalent in females and those with concurrent hypertension [19]. This study also found a significant association between hypertension and PVD, the association between sex and PVD was not explored.

Our study had certain limitations. Firstly, there were no control groups to compare the effect modification. Secondly, our study population mainly comprised elderly diabetics, and therefore we could not study the development of PVD in the younger population. Thirdly, no emphasis was made to determine the association of PVD with the duration of DM. And finally, since it was the first-ever study of its kind in our region, no comparative data could guide the association between risk factors and the PVD in diabetic patients.

Since this study was simply a descriptive cross-sectional study, it is recommended that a large-scale, randomized non-controlled trial be conducted in our region that should at least address the limitations in my research. This would help the investigators and clinicians determine the exact magnitude of PVD in this region and its associations with the common risk factors. Since PVD is more common in diabetic patients, this segment of the general population needs more attention as far as PVD is concerned. Therefore, a large-scale study will be valuable in addressing potential complications associated with the disease in this segment of the population.

## Conclusions

PVD is atherosclerotic blockage of vessels in lower limbs and is present in up to 14% of the general population. DM, however, is one of the most substantial risk factors for PVD and its associated complications. Our study showed a strong association of PVD with cigarette smoking, hypertension, hypercholesterolemia, and obesity. To decrease the risk of developing PVD, vigorous measures are needed to control these risk factors. Diabetic patients should be encouraged to quit cigarette smoking. They should be advised to maintain their high blood pressure if they are also diagnosed with hypertension through either salt restriction or antihypertensive medication. Problems of overweight, obesity, and hypercholesterolemia should be addressed accordingly to reduce the risk of the development of PVD.
